# Targeting the tyrosine kinase signalling pathways for treatment of immune-mediated glomerulonephritis: from bench to bedside and beyond

**DOI:** 10.1093/ndt/gfw336

**Published:** 2017-01-20

**Authors:** Terry King-Wing Ma, Stephen P. McAdoo, Frederick Wai Keung Tam

**Affiliations:** 1 Renal and Vascular Inflammation Section, Department of Medicine, Imperial College London, Hammersmith Hospital, Du Cane Road, London W12 0NN, UK; 2 Carol and Richard Yu Peritoneal Dialysis Research Centre, Department of Medicine and Therapeutics, Prince of Wales Hospital, Chinese University of Hong Kong, Sha Tin, Hong Kong

**Keywords:** crescentic glomerulonephritis, glomerulonephritis, IgA nephropathy, immunosuppression, lupus nephritis, tyrosine kinase

## Abstract

Glomerulonephritis (GN) affects patients of all ages and is an important cause of morbidity and mortality. Non-selective immunosuppressive drugs have been used in immune-mediated GN but often result in systemic side effects and occasionally fatal infective complications. There is increasing evidence from both preclinical and clinical studies that abnormal activation of receptor and non-receptor tyrosine kinase signalling pathways are implicated in the pathogenesis of immune-mediated GN. Activation of spleen tyrosine kinase (SYK), Bruton's tyrosine kinase (BTK), platelet-derived growth factor receptor (PDGFR), epidermal growth factor receptor (EGFR) and discoidin domain receptor 1 (DDR1) have been demonstrated in anti-GBM disease. SYK is implicated in the pathogenesis of ANCA-associated GN. SYK, BTK, PDGFR, EFGR, DDR1 and Janus kinase are implicated in the pathogenesis of lupus nephritis. A representative animal model of IgA nephropathy (IgAN) is lacking. Based on the results from *in vitro* and human renal biopsy study results, a phase II clinical trial is ongoing to evaluate the efficacy and safety of fostamatinib (an oral SYK inhibitor) in high-risk IgAN patient. Various tyrosine kinase inhibitors (TKIs) have been approved for cancer treatment. Clinical trials of TKIs in GN may be justified given their long-term safety data. In this review we will discuss the current unmet medical needs in GN treatment and research as well as the current stage of development of TKIs in GN treatment and propose an accelerated translational research approach to investigate whether selective inhibition of tyrosine kinase provides a safer and more efficacious option for GN treatment.

## INTRODUCTION

Glomerulonephritis (GN) affects patients of all ages and is an important cause of morbidity and mortality. It is estimated that there were >100 million prevalent cases of chronic kidney disease (CKD) secondary to GN globally in 2013, the number of which had increased by >30% since 1990 [1]. Immune-mediated glomerular injury plays an important role in the pathogenesis of anti-glomerular basement membrane (anti-GBM) disease, anti-neutrophil cytoplasmic antibody (ANCA)–associated glomerulonephritis (AAGN), lupus nephritis (LN) and immunoglobulin A nephropathy (IgAN). In recent years, advances in understanding the immunopathogenesis of these entities have provided translational opportunities for the development of novel therapeutic interventions [2].

Protein tyrosine kinases (PTKs) catalyze phosphorylation of tyrosine residues on protein substrates. They play a crucial role in the modulation of enzymatic activity and recruitment of downstream signalling molecules, which in turn regulate cellular growth and transformation [3]. PTKs can be classified into receptor tyrosine kinases (RTKs) and non-receptor tyrosine kinases (NRTKs). RTKs are transmembrane receptors that have intrinsic tyrosine kinase activity, whereas NRTKs are involved in different intracellular signalling pathways [4]. RTKs typically have an extracellular domain (for binding of different ligands), a transmembrane domain (for anchorage) and an intracellular domain (for signal transduction). Upon ligand binding to an RTK, it triggers dimerization and autophosphorylation of the receptor, followed by activation of various downstream signalling pathways [5]. NRTKs are subdivided into nine main families based on their similarities in domain structure. They interact with RTKs and mediate important signalling pathways that regulate cellular proliferative, differentiation, survival and apoptosis [6]. Dysregulation of PTK activity (e.g. overexpression) has been implicated in tumourigenesis, and the development of tyrosine kinase inhibitors (TKIs) has been one of the most important recent advances in oncology [7–9].

Recently there is increasing evidence from both preclinical and clinical studies that targeting tyrosine kinase signalling pathways is a potential therapeutic strategy for immune-mediated GN [10–13]. In this review we will focus our discussion on anti-GBM disease, AAGN, LN and IgAN. The potential clinical applications of TKIs in these conditions, their stage of development and preliminary results from clinical studies will be emphasized.

## CURRENT UNMET MEDICAL NEEDS

Rapidly progressive glomerulonephritis (RPGN) is an aggressive disease and the renal prognosis is often poor despite intensive treatment. A recent study from China showed that the 5-year the renal survival of anti-GBM disease and AAGN was 17.6 and 44.3%, respectively [14]. In another UK study of 43 patients (81% dialysis dependent at presentation), the 1-year renal survival of anti-GBM disease was just 16% [15]. AAGN usually affects elderly patients, and the use of non-selective immunosuppressive therapy can result in significant systemic side effects and sometimes fatal infectious complications. Rituximab (an anti-CD20 monoclonal antibody) is increasingly used in AAGN, but a recent study showed that there was no difference in clinical outcome of AAGN patients who were treated before and after the introduction of rituximab as an induction agent [16]. More importantly, the toxicity of rituximab was comparable to cyclophosphamide in the RAVE [17] and RITUXVAS [18] studies.

LN usually affects young female patients of child-bearing age. Some patients experience frequent relapses and require long-term immunosuppressive drugs. Corticosteroid-related systemic side effects and cyclophosphamide-related gonadal toxicity are important safety concerns. Multiple randomized controlled trials (RCTs) in ANCA-associated vasculitis (AAV) and LN have compared cyclophosphamide-based regimens with newer agents such as rituximab and mycophenolate mofetil. Disappointingly, their adverse event profiles were similar to those of cyclophosphamide-based protocols [19]. In high-risk IgAN patients with persistent proteinuria despite maximal supportive therapy and preserved renal function, the latest Kidney Disease: Improving Global Outcomes (KDIGO) guideline recommended immunosuppressive therapy using 6 months of corticosteroid [20]. However, the efficacy and safety of non-selective immunosuppressive treatment were recently challenged by the STOP-IgAN trial [21]. Compared with patients receiving supportive treatment alone, patients in the immunosuppression group had no significant improvement in the annual estimated glomerular filtration rate (eGFR) decline after 3 years but experienced significantly higher rates of severe infections, impaired glucose tolerance and weight gain.

With the current limitations of non-selective immunosuppressive therapy, a targeted approach using selective immunosuppressive drugs is more desirable and warrants further investigation.

## CURRENT LIMITATIONS OF TRANSLATIONAL RESEARCH IN GN

The essence of translational research is to make use of biomedical advances in basic science to address unmet medical needs of patients so as to improve patient outcomes [22]. In GN research, although various useful animal (mostly rodent) models of anti-GBM disease (e.g. experimental autoimmune GN, nephrotoxic nephritis), AAGN (e.g. experimental autoimmune vasculitis) and LN (e.g. lupus-prone mice) have been developed, none of them is perfect (Table 1) [23–25]. Development of an animal model of IgAN has been attempted, but none was sufficiently representative of human IgAN, partly attributed to the complex pathophysiology of IgAN [26]. This underscores the uncertainty of the predictive value of data from animal studies in human diseases. In the absence of a perfect animal model, immunohistochemistry (IHC) study of human renal biopsy becomes a valuable tool to provide additional evidence on the pathogenic role of a certain therapeutic target, assuming that the target protein is expressed in the kidney and not in circulating cells that regulate the autoimmune response. Using combined results from *in vitro* studies and IHC study of human renal biopsy may be a reasonable approach to provide a scientific basis for future clinical studies [27]. Various TKIs have been approved for the treatment of malignancy and have long-term efficacy and safety data in oncology patients. As a result, targeting the tyrosine kinase signalling pathways provides an attractive opportunity for accelerated translation research in GN treatment.

**Table 1.** Selected commonly used animal models of immune-mediated GN

Model	Resemblance of human disease	Animal	Method of induction	Advantages	Disadvantages
Experimental autoimmune GN (EAG)	Anti-GBM disease	Wistar Kyoto rat	Single intramuscular injection of collagenase-solubilized GBM (e.g. from Sprague-Dawley rat or sheep) in FCA or single intramuscular injection of recombinant rat α3(IV)NC1 in FCA	Invariable progression to chronic phase of injury which resembles human disease	Technically more demanding Some strains (e.g. Lewis rats) are resistant to EAG More gradual onset of disease compared with nephrotoxic nephritis
NTN	Anti-GBM disease	rat	Single intravenous injection of rabbit anti-GBM antisera	Relatively simple Rapid onset of renal injury	Rabbit antisera may contain antibodies towards other components apart from GBM
Accelerated nephrotoxic nephritis		Sprague-Dawley rat C57BL/6 mouse	Subcutaneous injection of sheep IgG in FCA followed by intravenous injection of sheep anti-rat/mouse GBM serum 5–10 days later	Rapid onset of renal injury	Variable progression to chronic phase of injury
Attenuated passive model of anti-GBM disease	Anti-GBM disease	C57BL/6 mouse	Intravenous injection of rabbit anti-mouse GBM antibody followed by intraperitoneal injection of purified mouse anti-rabbit IgG monoclonal antibody	Rapid onset of renal injury Degree of proteinuria is dependent on the amount of antibody used	Attenuated form of anti-GBM disease Only ∼50% of wild-type mice progressed to chronic phase
Passive anti-MPO transfer	ANCA-associated vasculitis	C57BL/6 wild-type or RAG2-deficient mice, with or without LPS priming	Anti-MPO antibody induced in MPO-deficient mice and transferred into recipients	Pauci-immune GN resembling human disease	Technically demanding Mild disease severity (reported crescent fraction 5–15%)
Experimental autoimmune vasculitis	ANCA-associated vasculitis	Wistar Kyoto rat	Immunization with human MPO in CFA	Dose-dependent effect of MPO on disease severity	Technically demanding for MPO purification No urinary abnormalities were seen in the other rat strains (BN, Lewis, WF)
Spontaneous mouse models of lupus nephritis	Lupus nephritis	MRL/*lpr* mouse	Spontaneous disease	A broad spectrum of SLE features including arthritis, inflammatory skin lesions and GN are seen	Nephritis is independent of FcγRs so the relevance to human lupus nephritis may not be totally appropriate
		NZB/NZW F1 mouse	Spontaneous disease	Closest approximation of human lupus nephritis in terms of characteristics of disease development and the underlying genetics driving autoimmunity	Slow onset of disease Progressive proteinuria beginning ∼5 months and azotemia ∼7 months onward
Anti-Thy 1.1 GN	Mesangial proliferative/IgAN	rat	Single intravenous injection of a mouse monoclonal anti-rat Thy 1.1 antibody	Mesangial cell proliferation and mesangial matrix expansion, histologically similar to human IgAN	No evidence of IgA deposition in glomeruli Lesions do not fully mimic the wide range of lesions seen in human IgAN
Spontaneous animal model for IgAN	IgAN	ddY strain mouse	Spontaneous disease	Elevated levels of circulating IgA and mouse IgA mesangial deposits, similar to human IgAN	Only a variable proportion of mice develop the disease model No haematuria and mild proteinuria
	IgAN	IgA1-expressing mouse	sCD89 injection	Mouse expressing both human IgA1 and CD89 have circulating and mesangial deposition of IgA1-sCD89 complexes resulting in kidney inflammation, haematuria and proteinuria	Issues with reproducibility Human IgA1 may not be representative of the pathogenetic IgA1 in patients

FCA, Freund's complete adjuvant; GBM, glomerular basement membrane; MPO, myeloperoxidase.

## EVIDENCE FROM PRECLINICAL STUDIES TO JUSTIFY FURTHER CLINICAL TRIALS OF TKIs IN IMMUNE-MEDIATED GN

### Anti-GBM disease

Compared with other types of immune-mediated GN, anti-GBM disease has been more extensively studied due to the availability of more robust animal models and it is considered a ‘prototypic’ autoimmune disease, such that findings may translate to other forms of GN.

Spleen tyrosine kinase (SYK) is an NRTK that plays a crucial role in a variety of biological functions, including intracellular signalling cascade for classic immunoreceptors like activatory Fc receptors (FcRs) and B-cell receptors (BCRs) [28]. IHC study showed increased SYK expression in both experimental [29–31] and human anti-GBM disease [32]. Increased SYK expression seemed to localize predominantly to areas of crescent formation and proliferating cells within the glomeruli [29, 32]. Administration of fostamatinib (an oral SYK inhibitor) completely aborted the development of nephritis when given before induction [29] and significantly reduced disease severity when given after established disease [29, 33]. In experimental autoimmune GN (EAG), fostamatinib treatment starting from Day 18 (where there were severe segmental necrotizing injury and crescent formation in ∼26% of glomeruli) to Day 36 led to a rapid and complete resolution of urinary abnormalities (100% reduction of both haematuria and proteinuria) that was sustained until Day 36 [29]. Fostamatinib-treated animals also had preserved levels of serum urea compared with a 103% increase in the vehicle group [29]. In nephrotoxic nephritis (NTN), high-dose fostamatinib treatment starting from Day 7 (where cellular crescents were present in ∼90% of glomeruli) to Day 14 significantly reduced proteinuria (23%), glomerular crescents (21%), infiltration of glomerular macrophages (93%) and CD8^+^ cells (74%) and serum creatinine (28%) [33]. SYK appeared to mediate glomerular injury by upregulation of pro-inflammatory cytokines, glomerular leukocyte recruitment and activation of c-Jun N-terminal kinase (JNK) and p38 mitogen-activated protein kinase (MAPK) pathways [30]. JNK inhibitor (CC-401) suppressed glomerular and tubulointerstitial damage when given before induction of experimental anti-GBM disease [34]. When given from Day 7 (where there was significant proteinuria, focal glomerular lesions, marked glomerular macrophage and T-cell accumulation and upregulation of pro-inflammatory mediators) to Day 14, CC-401 prevented renal impairment, suppressed proteinuria and prevented the development of severe glomerular and tubulointerstitial lesions, including crescent formation [35]. Pharmacological inhibition of p38 MAPKα/β, both early (1 h before induction) and late (starting from Day 4), have also been shown to be effective in reducing GN severity in NTN [36].

Bruton's tyrosine kinase (BTK) is an NRTK that plays an important role in signal transduction pathways that regulate B-cell survival, activation, proliferation and differentiation [37]. Activated SYK can induce phosphorylation of BTK, which cooperatively activates phospholipase C (PLC)-γ. PLC-γ catalyzes the hydrolysis of phosphatidylinositol 4,5-bisphosphate (PIP2) into diacylglycerol (DAG) and inositol 1,4,5-trisphosphate (IP3). IP3 induces calcium mobilization from the endoplasmic reticulum. DAG and calcium promote the activation of protein kinase C (PKC) and MAPK family downstream signalling cascades [38]. In experimental anti-GBM disease, administration of PF-06250112 (an oral BTK inhibitor) at the time of induction reduced proteinuria in a dose-dependent manner [39]. Interestingly, PF-06250112 inhibited disease development even in the presence of glomerular deposition of antibody and C3, indicating that the antiproteinuric effect was secondary to inhibition of the BTK signalling pathway instead of the effect on deposition or clearance of anti-GBM antibody. The effect of late treatment was not assessed in this study.

Platelet-derived growth factor receptors (PDGFRs) are RTKs that are expressed constitutively or inducibly in most renal cells. PDGFRs regulate cellular proliferation and migration, extracellular matrix accumulation, production of pro-inflammatory cytokines, tissue permeability and intrarenal haemodynamics [40]. PDGFR-β and PDGF-BB are overexpressed in the crescents of experimental and human anti-GBM disease [41]. An early study showed that intraperitoneal trapidil (a PDGFR antagonist) administration was associated with worse outcome *in vivo* [42]. However, recent studies using intraperitoneal imatinib (a multitargeted RTK inhibitor that can block PDGFR) showed significant renoprotective effects *in vivo*. In NTN, late imatinib treatment from Day 7 (where there was endocapillary proliferation, severe fibrinoid necrosis, cellular crescent formation and prominent glomerular fibrin deposition) to Day 20 led to less crescent formation and fibrinoid necrosis, reduced proteinuria and preserved renal function [43]. Using a similar NTN model, longer-term imatinib treatment from Day 7 to Day 49 significantly suppressed proteinuria, improved renal function and attenuated the development of glomerulosclerosis and tubulointerstitial injury [44]. In these *in vivo* studies, however, it was uncertain to what extent the beneficial effects were mediated specifically via inhibition of PDGFR signalling.

Epidermal growth factor receptor (EGFR) is an RTK that plays an important role in many cellular functions, including proliferation, migration and differentiation [45]. Heparin-binding epidermal growth factor-like growth factor (HB-EGF), a member of the EGFR family, is a potent inducer of cellular proliferation and migration (e.g. macrophages, T-lymphocytes). Upregulation of HB-EGF was found in both experimental and human anti-GBM disease [46]. HB-EGF deficiency status and pharmacological EGFR blockade (before induction) *in vivo* prevented renal leukocytic infiltration before the appearance of crescents and interstitial fibrosis, suggesting that the HB-EGF/EGFR pathway was involved in the very early stage of renal damage [46]. Pharmacological blockade of EGFR using erlotinib from Day 4 to Day 14 after induction of NTN was shown to reduce the expression of EGFR in the renal cortex, the proportion of crescentic glomeruli and blood urea nitrogen [46].

Discoidin domain receptor 1 (DDR1) is a collagen receptor with tyrosine kinase activity. As with most RTKs, MAPK and PI3 pathways are the downstream effectors of DDR1 [47]. DDR1 expression was increased in experimental and human anti-GBM disease [48]. DDR1-deficient mice had less severe renal disease and lower mortality than their wild-type littermates after induction of anti-GBM disease [49]. Administration of DDR1-specific antisense oligodeoxynucleotides at the time of induction decreased DDR1 expression and reduced disease severity. DDR1 antisense administration given on Day 4 (presence of proteinuria) and Day 8 both prevented progression of NTN, although the protective effect of the antisense treatment started at Day 8 was less efficient compared with antisense treatment started at Day 4 [49].

#### ANCA-associated GN

*In vitro* activation of neutrophil respiratory burst by ANCA from patients with systemic vasculitis required PTK and PKC activation. Blocking both kinases using pharmacological inhibitors abrogated ANCA-induced superoxide generation [50]. However, the specific tyrosine kinases involved were not investigated in this study. A previous study showed that p38 MAPK inhibition markedly reduced ANCA-induced neutrophil activation *in vitro* and partly reduced crescent formation *in vivo* [51].

SYK phosphorylation is induced during ANCA-triggered neutrophil activation [52]. In a study using the experimental autoimmune vasculitis model, where WKT rats developed haematuria and proteinuria at 4 weeks, fostamatinib treatment from Week 4 to Week 6 significantly reduced proteinuria, haematuria, glomerular histological abnormalities, glomerular macrophage infiltration, pulmonary haemorrhage severity and haemosiderin deposition in lung tissue [53]. Since SYK is involved in upstream signalling pathways of MAPK, the beneficial effect of SYK inhibition may be explained by its inhibitory effect on downstream MAPK signalling pathways. In patients with AAGN, glomerular SYK expression was increased and correlated with serum creatinine. SYK expression was highest in patients with crescentic GN (active disease) and minimal in those with sclerotic GN (chronic disease) [32].

In the kidney, vascular endothelial growth factor (VEGF) plays a crucial role in maintaining the integrity of the glomerular filtration barrier. Soluble fms-like tyrosine kinase 1 (sFlt-1) acts as an antagonist of VEGF. An imbalance of VEGF/sFlt-1 has been observed in many diseases with endothelial dysfunction, including diabetic nephropathy [54]. An *in vitro* study showed that ANCA antibodies increased sFlt1 during acute AAV, leading to an anti-angiogenic state that hinders endothelial repair [55].

#### Lupus nephritis

In prediseased lupus-prone NZB/NZW mice 6–7 months of age, fostamatinib treatment (up to Day 240) significantly delayed the onset of proteinuria and azotaemia, reduced renal inflammatory infiltrates and significantly prolonged animal survival [56]. In mice with established disease and proteinuria, fostamatinib treatment reduced proteinuria and preserved renal function in a dose-dependent manner and prolonged mice survival [56]. Up to 47% of mice with established disease demonstrated no microscopic evidence of renal changes after high-dose fostamatinib treatment, compared with only 10% in the vehicle group [56]. In MRL/*lpr* mice, fostamatinib treatment for 16 weeks starting from Week 4 (prediseased state) prevented the development of renal disease at Week 20, whereas fostamatinib for 8 weeks starting from Week 16 (established disease) significantly reduced proteinuria [57]. In a human renal biopsy study, patients with diffuse proliferative LN had the highest SYK expression, whereas those with membranous LN had minimal SYK expression [32]. Several BTK inhibitors have also been shown to reduce the severity of renal disease in experimental models of LN [13]. Ibrutinib treatment for 2 months in prediseased mice (starting from 4 months) alleviated renal damage and decreased circulating antinucleosome, antihistone and anti-ssDNA autoantibodies [58]. BTK inhibitors RN486 [59] and PF-06250112 [38] both reduced the severity of established GN in NZB/NZW mice.

In murine LN, imatinib treatment starting at 5 months of age (where focal glomerular hypercellularity and immune complex deposition were evident) significantly delayed the onset of proteinuria and renal impairment, protected against abnormal histological changes and prolonged animal survival, suggesting that inhibition of PDGFR might be a potential therapeutic strategy [60]. In another *in vivo* study using MRL/*lpr* mice, higher-dose imatinib treatment starting from Week 16 (advanced stage of GN) to Week 24 significantly reduced serum IgG and anti-dsDNA levels, ameliorated histological changes, reduced expression of PDGFR and transforming growth factor-β messenger RNA, reduced proteinuria, preserved renal function and prolonged survival [61]. An early IHC study showed increased EGFR expression in ∼35% of LN patients [62]. Autoantibodies to the extracellular domain of EGFR have been found in Fas-defective mice and in SLE patients [63]. A recent study showed that human epidermal growth factor receptor 2 (HER-2, an RTK) was overexpressed in lupus-prone NZM2410 mice and in patients with LN, but not in other mesangioproliferative GN [64]. DDR1 was found in podocytes and crescents in renal biopsies from patients with LN and genetic inhibition of DDR1 protected mice against development of crescentic GN [48].

Janus kinases (JAKs) are NRTKs that mediate the intracellular signalling initiated by interferons (IFNs), interleukins (ILs), colony-stimulating factors and hormones. Upon activation, JAKs phosphorylate the signal transducers and activators of transcription (STAT), which in turn regulate gene transcription. A series of JAK-STAT signalling cytokines, especially type I IFNs, IL-10 and IL-6, have been implicated in the pathogenesis of SLE [65]. Treatment of lupus-prone mice with JAK2 inhibitors led to prevention or improvement of established disease [66, 67]. In MRL/*lpr* mice, tyrphostin AG490 (a selective JAK2 inhibitor) treatment from Week 12 to Week 20 significantly inhibited renal expression of monocyte chemotactic protein (MCP)-1 and IFN-γ, reduced renal infiltration of T cells and macrophages, reduced proteinuria and improved renal function [66]. In an elegantly designed study, Lu *et al.* [67] tested the efficacy of CEP-33779 (a selective JAK2 inhibitor) in age-matched MRL/*lpr* or BWF1 mice with established SLE or LN, respectively. In this study, reference standard treatments including dexamethasone and cyclophosphamide were included. Treatment with CEP-33779 reduced serum pro-inflammatory cytokines and renal JAK2 activity, improved renal histopathology, decreased splenomegaly and lymphomegaly and prolonged animal survival. The therapeutic effect of CEP-33779 was comparable with that of cyclophosphamide and superior to dexamethasone alone. Tofacitinib, a JAK inhibitor, has been proven efficacious in rheumatoid arthritis. It is currently being investigated in a Phase I clinical trial of SLE patients (NCT02535689). Ruxolitinib, which inhibits JAK2, has been approved for the treatment of myelofibrosis. However, it has not been used in renal disease.

#### IgAN

Despite years of effort, a representative animal model of IgAN is still lacking. We [27] and others [68] have overcome this limitation by studying the effect of IgA1 purified from IgAN patients on human mesangial cells *in vitro*. In particular, we showed that IgA1 from patients with IgAN (but not IgA1 from the healthy volunteers) stimulated phosphorylation of SYK, production of inflammatory cytokines and growth factors and proliferation of mesangial cells *in vitro* [27]. These biological effects are similar to the pathological features of IgAN in patients. Inhibition of SYK by the active metabolite of fostamatinib or specific knockdown of SYK using siRNA reduced the synthesis of inflammatory cytokines and suppressed cell proliferation in IgA1-stimulated human mesangial cells [27]. In human IgAN, patients with endocapillary proliferation on renal biopsy had a higher level of SYK expression than those without [32].

Previous IHC study also showed that glomerular PDGFR-β expression significantly correlated with mesangial cell proliferation [69]. PDGFR inhibitor (in particular imatinib) and EGFR inhibitor reduced mesangial cell proliferation and matrix accumulation in rat acute anti-Thy 1.1 GN [40, 70]. In rat chronic anti-Thy 1.1 GN, PDGFR inhibition using B-specific oligonucleotide aptamer and neutralizing anti-PDGF-D IgG reduced proteinuria and improved renal function [40]. In acute anti-rat Thy-1.1, early erlotinib (an EGFR inhibitor) significantly prevented progression of mesangial cell proliferation and matrix accumulation and preserved renal function [41]. It should be noted, however, that the anti-rat Thy-1.1 GN model is not a representative model of human IgAN. In IgAN patients, elevated sFlt-1 (low VEGF:sFlt-1 ratio) correlated with the severity of proteinuria and hypertension [71]. Renal biopsy of IgAN patients also showed focal loss of VEGF in podocytes [72].

## POTENTIAL APPLICATIONS AND SAFETY CONCERNS OF TKIs IN IMMUNE-MEDIATED GN

TKIs are widely used clinically for the treatment of malignancies such as chronic myeloid leukemia (CML), gastrointestinal stromal tumors (GISTs), non-small-cell lung cancer and renal cell carcinoma. There is now accumulating evidence to suggest that further clinical studies of TKIs may be justified in selected immune-mediated GN (Table 2). Multiple *in vivo* studies have demonstrated beneficial effects of pharmacological inhibition of tyrosine kinases in established renal disease. Some of these tyrosine kinases are also upregulated in human renal biopsies. It should be noted, however, that the pathogenesis of anti-GBM disease and AVV are complex. Although targeting tyrosine kinase signalling pathways is attractive, it is unlikely that a single selective TKI can replace traditional induction therapy. Nevertheless, it might be reasonable to consider TKIs as adjunctive induction agents such that the dosage and side effects of non-selective immunosuppressive drugs may be reduced. Using TKIs as a steroid-sparing maintenance therapy may be another possible treatment strategy. In murine LN, JAK2 inhibitor was equally effective compared with cyclophosphamide [67]. The use of TKIs as induction and maintenance therapy in human LN might be justified.

**Table 2.** Summary of existing evidence of tyrosine kinase involvement in immunopathogenesis of immune-mediated GN

Tyrosine kinase	Disease	*In vitro* study	*In vivo* study	Human renal biopsy study	Justifiable for further clinical study
Spleen tyrosine kinase	Anti-GBM disease	√	√	√	√
	AAGN	√	√	√	√
	LN	√	√	√	√
	IgAN	√	No representative animal model	√	√
Bruton's tyrosine kinase	Anti-GBM disease	√	√	No data	Insufficient evidence
	AAGN	No data	No data	No data	Insufficient evidence
	LN	√	√	No data	Insufficient evidence
	IgAN	No data	No representative animal model	No data	Insufficient evidence
Platelet-derived growth factor receptor	Anti-GBM disease	√	Conflicting data	No data	Insufficient evidence
	AAGN	No data	No data	No data	Insufficient evidence
	LN	√	√	No data	Insufficient evidence
	IgAN	√	√ (in anti-Thy 1.1 model)	√	√
Epidermal growth factor receptor	Anti-GBM disease	√	√	√	√
	AAGN	No data	No data	No data	Insufficient evidence
	LN	√	√	√	√
	IgAN	√	√ (in anti-Thy 1.1 model)	No data	Insufficient evidence
Discoidin domain receptor 1	Anti-GBM disease	√	√	√	√
	AAGN	No data	No data	No data	Insufficient evidence
	LN	√	√	√	√
	IgAN	No data	No representative animal model	No data	Insufficient evidence
Janus kinase	LN	√	√	No data	Insufficient evidence
Vascular endothelial growth factor	IgAN	√	No representative animal model	√	√

Multiple TKIs have been approved for anti-cancer therapy. Imatinib was the first Bcr-Abl TKI approved by the US Food and Drug Administration for the treatment of CML. Imatinib also has inhibitory effects on other RTKs that make it a potent immunomodulatory agent. There have been promising results with the use of imatinib in murine models of kidney disease, including experimental anti-GBM disease, anti-Thy 1.1 GN and LN [73]. Besides, a number of case reports have described its successful (off-label) use in human membranoproliferative GN and cryoglobulinemia [74–76].

Although the clinical outcomes of these cases are encouraging, it should be noted that imatinib may have deleterious off-target effects on the kidney. In a recent long-term study of CML patients treated with different TKIs, imatinib was associated with a higher incidence of acute kidney injury (AKI) compared with dasatinib and nilotinib [77]. Imatinib-associated AKI has been reported previously [78]. It has also been associated with tubular dysfunction causing renal potassium and phosphate wasting [79] and thrombotic microangiopathy (TMA) [80]. Imatinib may also increase serum creatinine by inhibiting tubular secretion [81]. In another study of CML patients, patients with baseline renal dysfunction had a greater incidence of transient reversible AKI after dasatinib and nilotinib treatment [82]. Dasatinib has been reported to be associated with AKI [83, 84], thrombotic thrombocytopenia purpura [85] and nephrotic range proteinuria [86].

Fostamatinib has been evaluated in >3200 rheumatoid arthritis patients enrolled in three Phase 2, one Phase 2b and three Phase 3 trials [87, 88]. It is currently the only TKI that is being studied in a Phase 2, multicentre RCT in high-risk IgAN patients (NCT02112838). This clinical trial is testing a novel SYK-targeted approach for treating IgAN and will provide important information to guide further development of novel treatment strategies. Up to 35% of subjects on fostamatinib versus 11% on placebo developed hypertension or required adjustment to their antihypertensive regimen [89]. The effect of fostamatinib on blood pressure (mean elevation of ∼3 mmHg in both systolic and diastolic) appeared to be dose dependent and secondary to reduced VEGF-induced nitric oxide release from the endothelium [90]. This suggests that fostamatinib may also have off-target inhibitory effects on VEGF. Anti-VEGF therapy has been reported to be associated with hypertension, proteinuria and TMA [91]. However, previous trials of fostamatinib did not suggest an increased risk of nephrotoxic side effects. The current stages of development of TKIs in immune-mediated GN are summarized in Table 3.

**Table 3.** Stage of development of selected TKIs in immune-mediated GN

Drug	Target tyrosine kinase	Animal studies	Human studies
Fostamatinib	Spleen tyrosine kinase	Anti-GBM disease, ANCA-associated GN, lupus nephritis	Phase 2 clinical trial in IgAN
Ibrutinib	Bruton's tyrosine kinase	Lupus nephritis	No data
Imatinib	Platelet-derived growth factor receptor	Anti-GBM, lupus nephritis, anti-Thy 1.1 GN	Case reports of off-label use in membranoproliferative GN and cryoglobulinemia
Tofacitinib	Janus kinase	Lupus nephritis	Phase 1 clinical trial in SLE

## FUTURE DIRECTIONS

Clinical trials have been the Achilles' heel of translational nephrology. This is particularly true in the field of GN research. Some diseases (e.g. anti-GBM disease) are rare, and it is almost impossible to perform an RCT due to the long recruitment period and lack of statistical power of a study with a limited sample size. In this regard, establishing national or international patient registries in the field of rare diseases may be required. Despite numerous efforts, however, the development of novel treatment in these rare conditions remains difficult.

Another major difficulty in performing RCTs in nephrology is the definition of adequate surrogate end points. In many renal diseases (e.g. IgAN), the natural history is measured in terms of decades. While patients suffering from advanced cases should be recruited to get a sufficient number of events, patients with severe disease may be less responsive to therapy and experience more complications. In the recent STOP-IgAN trial, for instance, it has been challenging to give cyclophosphamide to patients with Stage 4 CKD, which may result in significant infective complications [21]. The relatively short duration of follow-up is another area of criticism, as a long-term renoprotective effect may not be apparent in the first few years, especially when the immunosuppression group has higher rate of proteinuria remission.

**FIGURE 1:**
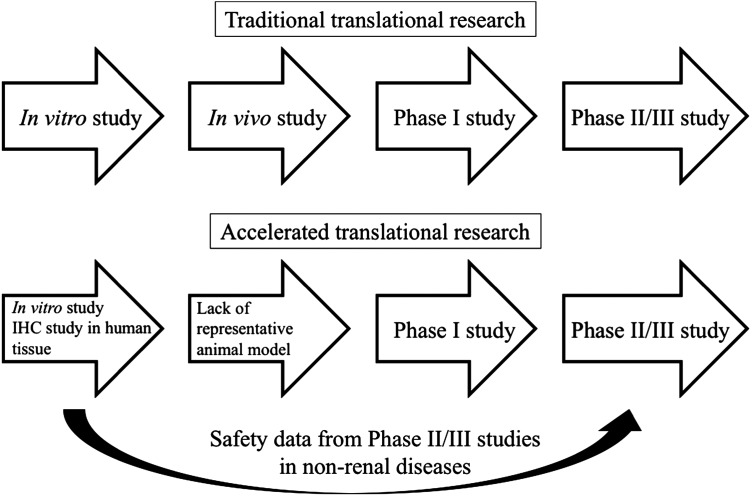
Schematic diagram showing a proposed accelerated pathway of translational research for the study of tyrosine kinase inhibitors in GN research.

The use of TKIs in selected immune-mediated GN appears to be supported by animal models and human biopsy studies. Observational studies or case series, despite a lack of randomization, have much lower costs and are relatively easy to perform. Provided that the nephrologist in charge has an adequate understanding of the pharmacology and potential side effects, off-label use of TKIs may be justified on a case-by-case basis after adequate explanation to patients with close monitoring. In some diseases where a representative animal model is lacking (e.g. IgAN), the development of a clinical trial may still be justified based on valid *in vitro* models and human renal biopsy data. Therefore, we propose an accelerated pathway of translational research for the study of TKIs in GN research (Figure 1).

## CONCLUSION

Targeting the tyrosine kinase signalling pathways represents a novel therapeutic target for the treatment of immune-mediated GN. Nonetheless, there is a persistent and even growing gap between advances in basic research and the development of clinical trials in GN research. Collaborations between scientists and clinicians are needed to address the current unmet medical needs and provide potential solutions to speed up translation into clinical practice and implementation of biomedical science advances.
